# Risk of gastric cancer in the second decade of follow-up after *Helicobacter pylori* eradication

**DOI:** 10.1007/s00535-019-01639-w

**Published:** 2019-10-30

**Authors:** Susumu Take, Motowo Mizuno, Kuniharu Ishiki, Chiaki Kusumoto, Takayuki Imada, Fumihiro Hamada, Tomowo Yoshida, Kenji Yokota, Toshiharu Mitsuhashi, Hiroyuki Okada

**Affiliations:** 1 Department of Internal Medicine, Fukuwatari Municipal Hospital, 1000 Fukuwatari, Takebe-cho, Kitaku, Okayama, 709-3111 Japan; 2 Department of Gastroenterology and Hepatology, Kurashiki Central Hospital, 1-1-1 Miwa, Kurashiki, Okayama, 710-8602 Japan; 3 Department of Internal Medicine, Nippon Kokan Fukuyama Hospital, 1840 Tsunoshita, Daimon-cho, Fukuyama, 721-0927 Japan; 4 Department of Surgery, Nippon Kokan Fukuyama Hospital, 1840 Tsunoshita, Daimon-cho, Fukuyama, 721-0927 Japan; 5 Department of Bacteriology, Okayama University Graduate School of Medicine Dentistry and Pharmaceutical Sciences, 2-5-1 Shikata-cho, Okayama, 700-8558 Japan; 6 Center for Innovative Clinical Medicine, Okayama University Hospital, 2-5-1 Shikata-cho, Okayama, 700-8558 Japan; 7 Department of Medicine and Medical Science, Okayama University Graduate School of Medicine Dentistry and Pharmaceutical Sciences, 2-5-1 Shikata-cho, Okayama, 700-8558 Japan

**Keywords:** *Helicobacter pylori*, Eradication therapy, Diffuse-type gastric cancer, Gastric atrophy

## Abstract

**Background and aims:**

Eradication of *Helicobacter pylori* reduces the risk of gastric cancer. In this study, we investigated the risk beyond 10 years after eradication of *H. pylori*.

**Methods:**

We conducted a retrospective cohort study of 2737 patients who had yearly endoscopic follow-up after cure of *H. pylori* infection. For comparison of gastric cancer risk in the second decade of follow-up with that in the first decade, we calculated standardized incidence ratios (SIRs) by dividing the number of observed cases of gastric cancer in the second decade of follow-up by that of expected cases which was estimated using the incidence rate ratio of age in the first decade.

**Results:**

During the follow-up for as long as 21.4 years (mean 7.1 years), gastric cancer developed in 68 patients (0.35% per year). The SIRs for diffuse-type gastric cancer was infinity (0 expected case and 4 observed cases) in patients with mild gastric mucosal atrophy and 10.9 (95% confidence interval 4.53–26.1) with moderate atrophy, whereas no significant increase of SIRs was observed in intestinal-type cancer regardless of the grade of baseline gastric atrophy or in diffuse-type cancer in patients with severe atrophy even though who had the highest risk.

**Conclusions:**

The longer the follow-up, the greater the risk of developing diffuse-type gastric cancer becomes in patients with mild-to-moderate gastric atrophy at baseline. Endoscopic surveillance should be continued beyond 10 years after cure of *H. pylori* irrespective of the severity of gastric atrophy.

## Introduction

*Helicobacter pylori* is a causative bacterium of several important upper gastrointestinal diseases such as peptic ulcer and gastric cancer [1, 2]. We have reported that eradication of *H. pylori* reduces the risk of developing gastric cancer [3, 4], and other reports have confirmed this finding [5–8]. Since we started *H. pylori* eradication therapy in 1995, more than 20 years have passed. During that period, we have reported results on several important medical issues through endoscopic follow-up of our cohort of patients who had received *H. pylori* eradication therapy [9–14]. Most recently, we have reported the incidence of Barrett’s adenocarcinoma after cure of *H. pylori* infection during the follow-up period of 20 years or more [15]. In the present study, we investigated the risk of gastric cancer developing more than 10 years after eradication of *H. pylori.* We also compared characteristics of this population with those whose cancer was detected less than 10 years, especially in relation to the background gastric mucosal atrophy and the histological type of cancer.

## Patients and methods

We recruited 3450 consecutive patients who had received successful eradication therapy for *H. pylori* infection at the outpatient clinic of Nippon Kokan Fukuyama Hospital from June 1995 to July 2015. We excluded 713 patients who had not had follow-up endoscopy more than one year (*n* = 668), had gastric cancer or Barrett’s adenocarcinoma diagnosed within one year follow-up (*n* = 29); or had positive urea breath tests at one year follow-up, suggesting misjudgment of successful eradication (*n* = 16). The remaining 2737 patients were enrolled. There were 544 women and 2193 men, with a mean age of 53.8 years (range 20.7–81.4 years). The patients were mostly male factory workers at JFE Steel Corporation, West Japan Works. The patients had undergone endoscopic examination before receiving eradication therapy to evaluate peptic ulcers, background gastric mucosal atrophy and status of *H. pylori* infection; we documented that none of patients had gastric cancer then. Among the 2737 patients, 1198 had gastric ulcer, 865 had duodenal ulcer, 136 had both diseases, and 538 had chronic gastritis but no ulcer.

Gastric mucosal atrophy was evaluated according to the endoscopic–atrophic-border scale described by Kimura and Takemoto [16, 17], which correlates with the results of histological evaluation [18] and was classified by degree into three grades: mild (C-1 and C-2), moderate (C-3 and O-1), or severe (O-2 and O-3). Patients were excluded if they had previously undergone gastrectomy or received endoscopic therapy for gastric neoplasms; were pregnant; had an allergy to penicillin, clarithromycin or metronidazole; were taking anticoagulants; or had used a proton pump inhibitor, H_2_ receptor antagonist, adrenocortical steroids, or non-steroidal anti-inflammatory drugs within the month preceding the eradication therapy.

### Eradication therapy and post-eradication schedule

*Helicobacter pylori* infection was defined as a positive bacterial culture from endoscopic biopsy specimens taken before eradication therapy was begun. The specimens were obtained from the greater curvature of the body and the antrum of the stomach and cultured with Brucella agar containing 7% horse blood and antibiotics. Urease activities of the specimens were tested in a modified rapid urease test (MR UREA S; Institute of Immunology Co., Tokyo, Japan).

Patients received *H. pylori* eradication therapy as described [3]. One to 2 months and 1 year after the completion of therapy, including the cessation of maintenance therapy with acid secretion inhibitors, a ^13^C-urea breath test and endoscopy were carried out in each patient to determine *H. pylori* status and to reconfirm that there was no gastric cancer. *H. pylori* infection was considered cured when the urea breath test (cut-off value, 3.5 per mil) [19] was negative. After confirmation of eradication and the absence of upper gastrointestinal tract neoplasm, the patients underwent follow-up endoscopy yearly.

Gastric cancer was classified according to Lauren [20] and Ueyama et al. [21] as intestinal type, diffuse type, or gastric adenocarcinoma of fundic gland type. For analysis, the fundic gland type cancer was included in the intestinal type cancer. The study was conducted according to the guidelines of the Declaration of Helsinki. A local ethics committee approved the study protocol. The objective of the study was explained to all patients before their participation, and written informed consent was obtained from each patient.

### Statistical analysis

Survival curves were constructed by the Kaplan–Meier method. For comparison of the risk of gastric cancer in the second decade of follow-up with that in the first decade after cure of *H. pylori* infection, we calculated the standardized incidence ratio (SIR) of gastric cancer by dividing the number of observed cases of gastric cancer in the second decade of follow-up by that of expected cases in the second decade, which was estimated using the incidence rate ratio of age in the patients during the first decade of follow-up. For age adjustment, we used the incidence rate ratio of age obtained by Poisson regression analysis. Patients’ ages in relation to grade of background gastric mucosal atrophy at eradication therapy were analyzed using linear regression analysis with age as the response variable and gastric mucosal atrophy as the explanatory variable, using mild gastric atrophy as a reference. The other characteristics were analyzed by Chi-square test.

## Results

Baseline characteristics of the 2737 patients who received successful eradication therapy are provided in Table 1. Patients were followed for up to 21.4 years (mean 7.1 years) until June 2017. Positive urea breath tests, suggesting possible reinfection with *H. pylori*, were noted in 24 patients 7.3 ± 3.8 years (mean ± standard deviation) after the start of follow-up; analysis of these patients was stopped at the time of the possible reinfection.

**Table 1:** Patients’ demographic characteristics

Characteristics	*n* = 2737
Gender	
Male	2193
Female	544
Age (year)	53.8 ± 9.1
Smoking	
Absence	1390
Presence	1347
Drinking	
Absence	1189
Presence	1548
Ulcer	
No ulcer	538
Duodenal ulcer	865
Gastric ulcer	1198
Both	136
Background mucosal atrophy^a^	
Mild	801
Moderate	1090
Severe	846
Duration of follow-up (years)	7.1 ± 5.4
Eradication therapy^b^	
Dual	445
Triple	1962
Metronidazole-based therapy	330
*H. pylori* reinfection	
Absent	2713
Present	24

^a^Gastric mucosal atrophy was evaluated according to the endoscopic-atrophic-border scale and was classified by grades; mild (C-1 and C-2), moderate (C-3 and O-1), or severe (O-2 and O-3) at eradication therapy as described in the text

^b^*Helicobacter pylori* eradication therapy: dual, a proton pump inhibitor together with amoxicillin; triple, a proton pump inhibitor together with amoxicillin and clarithromycin; metronidazole, a proton pump inhibitor together with metronidazole and amoxicillin or clarithromycin

**Fig. 1:**
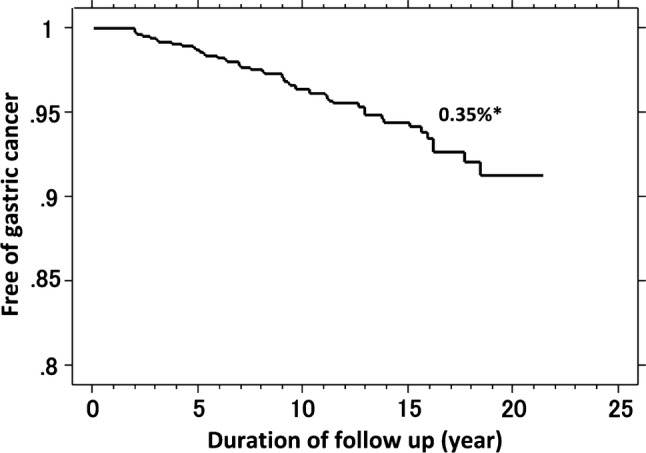
Kaplan–Meier analysis of the proportion of patients who remained free of gastric cancer after cure of *H. pylori* infection. Asterisk: the annual risk of developing gastric cancer calculated by Kaplan–Meier analysis

**Fig. 2:**
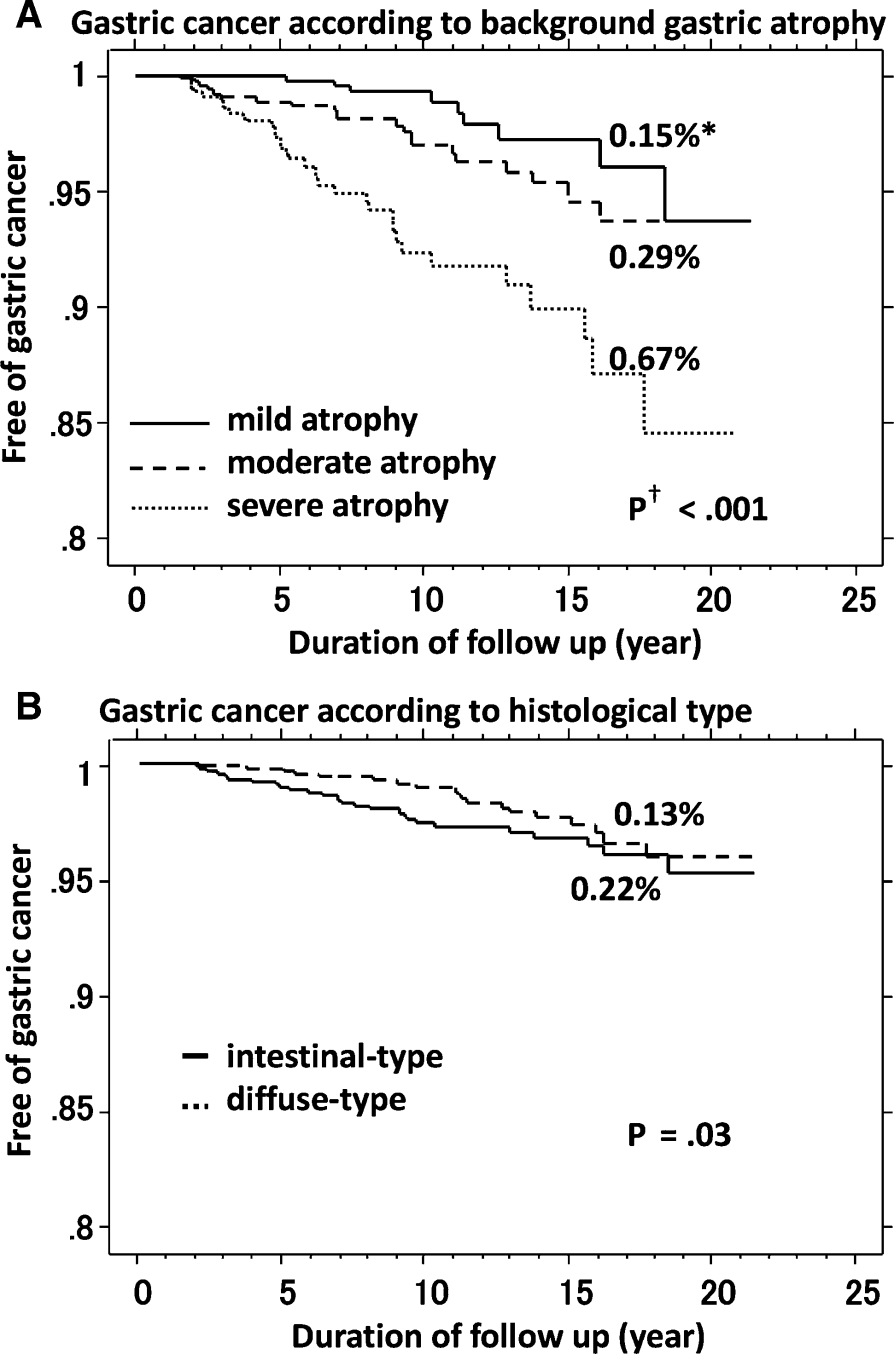
Kaplan–Meier analysis of the proportion of patients who remained free of gastric cancer after cure of *H. pylori* infection according to grade of background gastric mucosal atrophy at the time of eradication therapy (**a**) and histological type of gastric cancer (**b**) during the total follow-up period as long as 21 years. Asterisk: the annual risk of developing gastric cancer calculated by Kaplan–Meier analysis. Dagger: statistical significance between curves tested by log-rank test

During the follow-up period, gastric cancer developed in 68 of the 2737 patients. Endoscopic features [22], TNM stages, and histological types of the gastric cancers are presented in Table 2. Most cancers were endoscopically superficial type (55/68, 81%) and in the early TNM stage (60/68, 88%). Histologically, 43 of the cancers were intestinal type (including 6 fundic gland-type gastric cancer), and 25 were diffuse type. With regard to the prognosis, all-cause and disease-specific mortality occurred in 9 and 4 patients, respectively, among the 68 patients during the follow-up period. By Kaplan–Meier analysis (Fig. 1), the risk for all gastric cancer after cure of *H. pylori* infection was 0.35% per year. The longest interval between *H. pylori* eradication and the occurrence of cancer was 18.3 years. Figure 2 presents Kaplan–Meier analysis of the proportion of patients who remained free of gastric cancer during the total follow-up period (for as long as 21 years after cure of *H. pylori* infection) according to grade of background gastric mucosal atrophy at the time of eradication therapy and histological type of gastric cancer. Gastric cancer developed in 9 of 801 patients who had mild atrophy (0.15% per year), 23 of 1090 who had moderate atrophy (0.29% per year), and in 36 of 846 who had severe atrophy (0.67% per year) (*P* ≤ 0.001, log-rank test) (Fig. 2a). The annual rate for developing intestinal-type cancer was 0.22% and the rate for diffuse-type was 0.13% (*P* = 0.03) (Fig. 2b).

**Table 2:** Characteristics of 68 gastric cancers that developed after *H. pylori* eradication

	*N*
Endoscopic features^a^	
Superficial depressed (0 − IIc)	36
Superficial depressed + elevated (0 − IIc + IIa)	4
Superficial flat type (0 − IIb)	12
Superficial elevated type (0 − IIa)	3
Depressed type (0 − III)	3
Submucosal tumor-like	2
Borrmann’s classification type 2	4
Borrmann’s classification type 3	2
Borrmann’s classification type 4	2
TNM stages	
Stage IA	58
Stage IB	2
Stage IIA	4
Stage IIB	1
Stage IIIA	2
Stage IV	1
Histological type	
Intestinal type^b^	43
Diffuse type	25

^a^Endoscopic features according to classification of Japanese Gastric Cancer Association [22]

^b^Six fundic gland-type cancers were classified into intestinal-type cancer

**Fig. 3:**
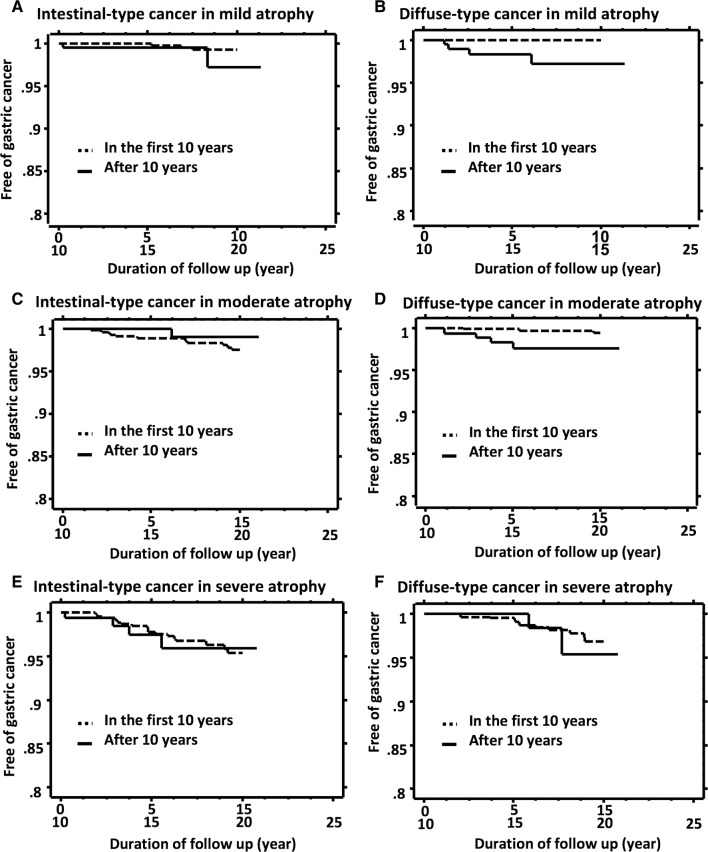
Kaplan–Meier analysis of the proportion of patients who remained free of gastric cancer after cure of *H. pylori* infection according to grade of background gastric mucosal atrophy at the time of eradication therapy, histological types of gastric cancer, and follow-up period. Dotted line: Kaplan–Meier curve of patients free of gastric cancer in the first 10 years after cure of *H. pylori* infection. Line: Kaplan–Meier curve of patients free of gastric cancer in the second decade of follow-up, which was created from the 10 years as starting point

When we carefully examined the Kaplan–Meier analysis, we noticed a change of slopes in certain groups as the interval after *H. pylori* eradication became longer. Thus, we arbitrarily created Kaplan–Meier curves of patients free of gastric cancer in the second decade of follow-up from the 10 years as starting point and overlapped them on the curves in the first 10 years after cure of *H. pylori* infection (Fig. 3). In intestinal-type gastric cancer, the slopes in the second decade of follow-up were like those in the first decade among patients with each grade of background atrophy (Fig. 3a, c, e). In contrast, in diffuse-type gastric cancer, the slopes in the second decade of follow-up in patients with mild gastric atrophy and moderate atrophy were steeper than those in the first decade among patients with corresponding grade of background gastric mucosal atrophy (Fig. 3b, d), whereas the slopes were similar in patients with severe atrophy (Fig. 3f).

To statistically analyze the observed changes of the slopes in the second decade of follow-up, we calculated the SIRs of gastric cancer according to grade of background gastric atrophy at the time of eradication therapy and histological types of gastric cancer (Table 3). In total gastric cancer, SIR was increased in patients with mild gastric atrophy but not in those with moderate or severe atrophy. The SIRs for diffuse-type gastric cancer were infinity (0 expected case and 4 observed cases; 95% confidence of interval, not applicable) in patients with mild gastric atrophy and 10.9 (95% confidence interval 4.53–26.1) in patients with moderate atrophy, whereas no significant increase of SIRs was present in intestinal-type cancer, regardless of the grade of baseline gastric mucosal atrophy or in diffuse-type cancer in patients with severe gastric atrophy.

**Table 3:** The standardized incidence ratio of gastric cancer in the second decade after *H. pylori* eradication according to grade of background gastric atrophy at the time of eradication therapy and histological types of gastric cancer

	Mild^a^*n* = 801				Moderate *n* = 1090				Severe *n* = 846			
	Observed^b^	Expected^c^	SIR	95% CI	Observed	Expected	SIR	95% CI	Observed	Expected	SIR	95% CI
Total cancer	6	1.23	4.89	2.19–10.8	6	5.40	1.11	0.50–2.47	6	7.29	0.82	0.37–1.83
Intestinal^d^	2	1.71	1.17	0.29–4.68	1	6.26	0.16	0.02–1.13	4	5.25	0.76	0.29–2.03
Diffuse	4	0	∞	n/a	5	0.46	10.9	4.53–26.1	2	2.12	0.94	0.24–3.77

*SIR* the standardized incidence ratio of gastric cancer in the second decade of follow-up calculated by dividing the number of observed cases of gastric cancer by that of the expected cases, *CI* confidence interval, *n/a* not applicable

^a^Grade of background gastric mucosal atrophy at the time of *H. pylori* eradication

^b^Numbers of gastric cancer cases observed in the second decade of follow-up

^c^Numbers of expected gastric cancer cases in the second decade of follow-up estimated using the incidence rate ratio of age in the patients during the first decade of follow-up. For age adjustment, we used the incidence rate ratio of age obtained by Poisson regression analysis

^d^Histological type of gastric cancer; six fundic gland type cancers were classified into intestinal-type cancer

Because the risk of developing gastric cancer was closely related to the background gastric mucosal atrophy at baseline, we compared the patients’ demographics in relation to the grade of atrophy (Table 4). Mean ages at the time of eradication therapy increased in relation to the severity of gastric atrophy (50.6 ± 9.1 years, 53.7 ± 8.7, and 57.0 ± 8.5 in mild, moderate, and severe atrophy, respectively) (linear regression analysis, *P* < 0.001). Female patients less often had severe atrophy and more often had mild atrophy than did male patients (*P* < 0.01, Chi-square test, residual analysis). Patients with peptic ulcer disease were more likely to have mild atrophy and less likely to have severe atrophy (*P* < 0.01). Cigarette smoking or alcohol consumption was not related to the grade of mucosal atrophy.

**Table 4:** Patients’ demographic characteristics in relation to grade of background gastric mucosal atrophy at eradication therapy

	Total	Gastric mucosal atrophy^a^		
		Mild	Moderate	Severe
	*N* = 2737	*N* = 801	*N* = 1090	*N* = 846
Age^b^	53.8 ± 9.1	50.6 ± 9.1	53.7 ± 8.7	57.0 ± 8.5
Gender^c^				
M	2193	629	844	720
F	544 (19.9%)	172 (21.5%)	246 (22.6%)	126 (14.9%)
Drinking^d^				
Absence	1189	348	495	346
Presence	1548 (56.6%)	453 (56.6%)	595 (54.6%)	500 (59.1%)
Smoking^d^				
Absence	1390	410	554	426
Presence	1347 (49.2%)	391 (48.8%)	536 (49.2%)	420 (49.6%)
Peptic ulcer^e^				
Absence	538	95	200	243
Presence	2199 (80.3%)	706 (88.1%)	890 (81.7%)	603 (71.3%)

^a^Gastric mucosal atrophy was evaluated according to the endoscopic-atrophic-border scale as described in the text

^b^In the linear regression analysis with age as the response variable and gastric mucosal atrophy as the explanatory variable using mild atrophy as a reference, the regression coefficient (95% confidence interval) was 3.11 (2.31–3.90, *P* < 0.001) in moderate atrophy and 6.43 (95% 5.59–7.28, *P* < 0.001) in severe atrophy

^c^Female gender was less common in patients with severe atrophy than those with mild or moderate atrophy (*P* < 0.01, Chi-square test, residual analysis)

^d^Smoking or drinking habit was not significantly different among the patients’ groups

^e^Peptic ulcer diseases were less common in patients with severe atrophy than those with mild or moderate atrophy (*P* < 0.01, Chi-square test, residual analysis)

## Discussion

In the present study, when we extended the follow-up period after eradication of *H. pylori* as long as 21 years, we found that the risk of developing diffuse-type gastric cancer became greater in the second decade of follow-up than during the first 10 years. This increase was only found in patients with mild-to-moderate gastric atrophy at baseline. One might expect that the gastric cancer risk is minimal when *H. pylori* is eradicated particularly in persons without significant gastric mucosal atrophy [11, 13]. This expectation was true at 10 years but was not true for diffuse-type gastric cancer after more than 10 years follow-up.

The risk of gastric cancer did not change between the first and the second decade of follow-up for intestinal-type gastric cancer regardless of the grade of baseline gastric mucosal atrophy. Patients with severe gastric mucosal atrophy at baseline did not have any increase in the second decade of follow-up for either histological type of gastric cancer. Thus, the increase of gastric cancer risk after 10 years follow-up appeared to be specific to diffuse-type gastric cancer in patients without significant gastric mucosal atrophy at the time of eradication therapy. Our findings suggest that the longer the follow-up for more than 10 years after *H. pylori* eradication, the greater the risk of developing diffuse-type gastric cancer becomes in patients with mild-to-moderate gastric atrophy at baseline. It should be appreciated that even though the rate of gastric cancer developing in patients with severe mucosal atrophy did not increase in the second decade after *H. pylori* eradication, severe atrophy continued to be a risk factor for gastric cancer, as shown in Fig. 3.

Regarding risk factors for developing gastric cancer, age is one of the important risk factors. The baseline age at *H. pylori* eradication was less in patients with mild or moderate than with severe gastric atrophy in our cohort, but the risk did not change in case of intestinal-type cancer through the follow-up period up to 21 years regardless of the grade of baseline mucosal atrophy, a finding suggesting that factors other than aging are involved. Early gastric cancers of diffuse type are often superficial and flat, with only discoloration and are difficult to recognize endoscopically [23]. During the past decades, endoscopy equipment has improved greatly, and techniques such as magnifying endoscopy with narrow band imaging have improved the ability to detect early diffuse-type gastric cancer [24]. Thus, the apparent increase in the incidence of gastric cancer during the second decade of follow-up might be due to the availability of better instruments. However, in patients with severe gastric atrophy, the rate of diffuse-type cancer did not change in the second decade of follow-up, implying that better instruments cannot fully explain the increase of risk selectively for diffuse-type gastric cancer in patients with mild-to-moderate mucosal atrophy. In reported word [25], a comprehensive molecular evaluation of gastric cancer revealed distinct and salient differences of genomic features between gastric cancer of the intestinal and diffuse types. Such differences, aging, and/or other environmental factors might have influenced the effect of *H. pylori* eradication on the development of the histological types of gastric cancer and could have played a role in the change of the risk of diffuse-type cancer more than 10 years after *H. pylori* eradication. Further investigation is needed to explore these genomic issues.

A recent report of an analysis of a large database in Hong Kong described that a decrease in the risk of gastric cancer was more prominent more than 10 years after *H. pylori* eradication therapy among people aged 40–59 years and even over 60 years [7]. That finding appears discordant with the results of the present study. Many differences, such as study methods and subjects involved, might be responsible for the differences.

A major implication from the results of this study is that endoscopic surveillance of patients cured of *H. pylori* should be continued beyond 10 years, even for those without severe atrophic gastritis. This policy should apply to patients with mild gastritis, whom we had considered at minimal risk for developing gastric cancer after eradication of *H. pylori*, as their risk of developing cancer even increased after 10 years, mainly due to increase of the risk for diffuse-type cancer. An advantage of continuing the endoscopic surveillance also is that it can detect gastric cancer at early stage as well as detecting esophageal cancer [15]. In addition, we should be aware of diffuse-type gastric cancer at surveillance, understanding its endoscopic features when follow-up period after *H. pylori* eradication becomes longer. In a recent retrospective study of early gastric cancer detected after eradication of *H. pylori* and treated with endoscopic resection, there was a higher rate of gastric cancer with mild atrophy at the time of detection in cancers found after 5 years follow-up than that during the first 4 years [26]. Because the significant differences of study designs, subjects, and the timing of evaluation of background gastric atrophy, it is not clear how their findings relate to ours.

Strengths of our study are these: (1) the study is the largest we are aware of on the long-term follow-up of patients treated for eradication of *H. pylori*; (2) data were collected through the prospective cohort study with scheduled endoscopic follow-up; (3) *H. pylori* status was evaluated during the follow-up, and possible influence of reinfection or recrudescence of *H. pylori* was excluded. Limitations of our study are these: (1) The study was an observational analysis performed in a single hospital. (2) Patients were mostly male patients with peptic ulcer diseases. People with *H. pylori* infection have chronic gastritis, but peptic ulcer diseases do not develop in most of them. Whether the findings of this study apply to people without peptic ulcers should be evaluated. (3) The possible contribution of temporal change of technology and knowledge was not evaluated; impact of use of better instruments and accumulation of understandings of endoscopic features of early gastric cancer on our findings was uncertain.

In conclusion, long-term endoscopic follow-up of patients cured of *H. pylori* infection revealed that the risk of gastric cancer developing after eradication of *H. pylori* was greater in the second decade of follow-up than in the previous 10-year period. The increased risk was specific to diffuse-type gastric cancer among patients with mild-to-moderate gastric atrophy. These findings indicate that endoscopic surveillance for gastric cancer should be continued beyond 10 years after cure of *H. pylori* infection irrespective of severity of gastric mucosal atrophy at eradication therapy.
